# Paving Plant-Food-Derived Bioactives as Effective Therapeutic Agents in Autism Spectrum Disorder

**DOI:** 10.1155/2021/1131280

**Published:** 2021-08-21

**Authors:** Natália Cruz-Martins, Cristina Quispe, Celale Kırkın, Ezgi Şenol, Aslı Zuluğ, Beraat Özçelik, Adedayo O. Ademiluyi, Olubukola Helen Oyeniran, Prabhakar Semwal, Manoj Kumar, Farukh Sharopov, Victor López, Francisco Les, Iulia-Cristina Bagiu, Monica Butnariu, Javad Sharifi-Rad, Mohammed M. Alshehri, William C. Cho

**Affiliations:** ^1^Faculty of Medicine, University of Porto, Alameda Prof. Hernâni Monteiro, 4200-319 Porto, Portugal; ^2^Institute for Research and Innovation in Health (i3S), University of Porto, 4200-135 Porto, Portugal; ^3^Institute of Research and Advanced Training in Health Sciences and Technologies (CESPU), Rua Central de Gandra, 1317, 4585-116, Gandra, PRD, Portugal; ^4^Facultad de Ciencias de la Salud, Universidad Arturo Prat, Avda. Arturo Prat 2120, Iquique 1110939, Chile; ^5^Department of Food Engineering, Faculty of Chemical and Metallurgical Engineering, Istanbul Technical University, Maslak, 34469 Istanbul, Turkey; ^6^Department Food Engineering, Faculty of Engineering and Natural Sciences, Istanbul Sabahattin Zaim University, Beyoglu, 34427 Istanbul, Turkey; ^7^Department of Gastronomy and Culinary Arts, School of Applied Sciences, Ozyegin University, Cekmekoy, 34794 Istanbul, Turkey; ^8^Department Food Engineering, Faculty of Chemical and Metallurgical Engineering, Istanbul Technical University, Maslak, 34469 Istanbul, Turkey; ^9^BIOACTIVE Research & Innovation Food Manufacturing Industry Trade Ltd. Co., Maslak, Istanbul 34469, Turkey; ^10^Functional Foods, Nutraceuticals, and Phytomedicine Unit, Department of Biochemistry, Federal University of Technology, Akure 340001, Nigeria; ^11^Department of Biotechnology, Graphic Era University, Dehradun, Uttarakhand, India; ^12^Uttarakhand State Council for Science and Technology, Dehradun, Uttarakhand, India; ^13^Chemical and Biochemical Processing Division, ICAR - Central Institute for Research on Cotton Technology, Mumbai 400019, India; ^14^Department of Pharmaceutical Technology, Avicenna Tajik State Medical University, Rudaki 139, 734003 Dushanbe, Tajikistan; ^15^Facultad de Ciencias de la Salud, Universidad San Jorge, Villanueva de Gállego, Zaragoza, Spain; ^16^Instituto Agroalimentario de Aragón (IA2), Universidad de Zaragoza-CITA, Zaragoza, Spain; ^17^Victor Babes University of Medicine and Pharmacy of Timisoara, Department of Microbiology, Timisoara, Romania; ^18^Multidisciplinary Research Center on Antimicrobial Resistance, Timisoara, Romania; ^19^Banat's University of Agricultural Sciences and Veterinary Medicine “King Michael I of Romania” from Timisoara, Timisoara, Romania; ^20^Phytochemistry Research Center, Shahid Beheshti University of Medical Sciences, Tehran, Iran; ^21^Pharmaceutical Care Department, Ministry of National Guard-Health Affairs, Riyadh, Saudi Arabia; ^22^Department of Clinical Oncology, Queen Elizabeth Hospital, Kowloon, Hong Kong

## Abstract

Autism spectrum disorder (ASD) is a neurodevelopmental disorder, where social and communication deficits and repetitive behaviors are present. Plant-derived bioactives have shown promising results in the treatment of autism. In this sense, this review is aimed at providing a careful view on the use of plant-derived bioactive molecules for the treatment of autism. Among the plethora of bioactives, curcumin, luteolin, and resveratrol have revealed excellent neuroprotective effects and can be effectively used in the treatment of neuropsychological disorders. However, the number of clinical trials is limited, and none of them have been approved for the treatment of autism or autism-related disorder. Further clinical studies are needed to effectively assess the real potential of such bioactive molecules.

## 1. Introduction

**Figure 1:**
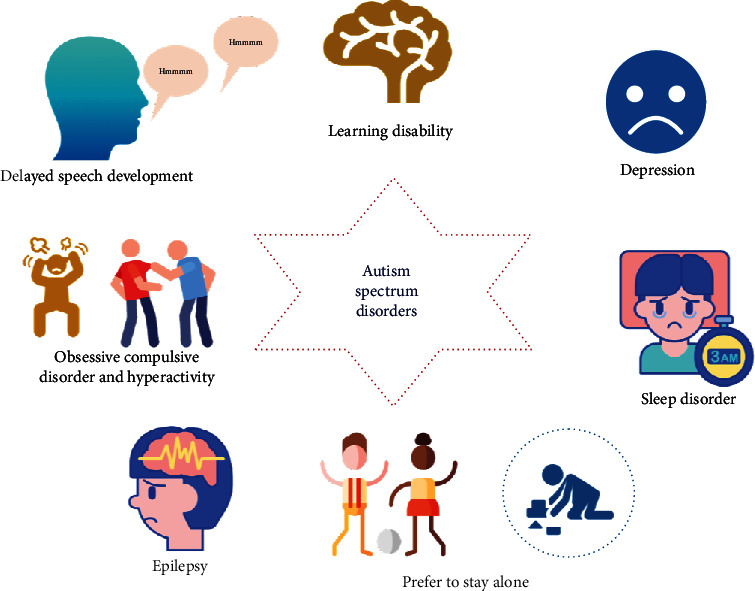
Illustration showing autism spectrum diseases (ASD).

Autism spectrum disorder (ASD) is a set of behavioral and neurodevelopmental diseases featuring social and communication deficits accompanied by increased repetitive and/or restrictive behaviors [1]. The most commonly registered behaviors under ASD are presented in Figure 1. In the last years, the prevalence of ASD has dramatically risen [2], and as per the World Health Organization (WHO), it is estimated that 1 in 160 children in the world has ASD [3]. Although the etiology and pathogenesis of this disorder are not fully understood, several environmental and genetic factors have been proposed as mediators, thus, limiting key molecular mediators' identification as well as possible neurological and biochemical mechanisms. However, recent evidences are pointing to alterations in hormones, amino acids, and several biochemical markers as possible mediators in autistic individuals [4, 5]. Furthermore, unifying etiology involving immune dysfunction, abnormal lipid metabolism, glutamatergic dysfunction, and raised susceptibility to oxidative stress has been proposed for ASD [6], with studies increasingly linking mitochondrial dysfunction to ASD [1, 7, 8]. Indeed, mitochondrial dysfunction and oxidative metabolism defects are characteristic of many chronic illnesses, such as bipolar disorder, multiple sclerosis, Parkinson's disease (PD), schizophrenia, depression, ASD, and chronic fatigue syndrome [9]. Since mitochondria is an integral part of many cell processes, it becomes susceptible to many insults that could affect its integrity. Furthermore, evidences are pointing at immune dysregulation in ASD etiology [9]. Several studies have revealed the presence of immune abnormalities in those suffering from autism [10, 11], with markers of autoimmunity, abnormal cell immunity, aberrant expression of cytokines, and other soluble immunity mediators being evident in ASD children [10–13].

Nonetheless, faced by data scarcity on ASD etiology and underlying causes, the ability to develop and mobilize effective treatments is still limited. Hence, efforts have been done on alleviating only comorbid manifestations of the disorder [1]. However, as a result of the limited treatment options available to improve ASD symptoms, financial challenges, and drugs side effects, dietary and nutritional approaches are becoming popular components of ASD management [14, 15]. In this sense, the present review is aimed at providing a brief overview on ADS and related pathophysiology, as well as on the promissory therapeutic abilities evidenced by plant-food bioactives.

## 2. Autism Spectrum Disease: A Brief Overview

ASD is an increasingly frequent disorder found among children. To date, several risk factors (prenatal, perinatal, and postnatal) that are either genetic or environmental have been stated [16]. Approximately, 15% cases of ASD are linked to genetic disorders (fragile X syndrome, neurofibromatosis, tuberous sclerosis, and Rett syndrome) [17], and various genetic alterations, such as de novo mutations in coding regions, copy number variations, and chromosomal alterations, are the most frequent variants that have been associated with ASD development [18, 19]. The significance of genetic factors in ASD development could be easily explained in twin studies, with results from these studies indicating that the ASD concordance rates could be around 30–99% in monozygotic twins, 0–65% in dizygotic twins, and 3–30% in siblings, respectively [20–23], while a recent study showed a lower ASD concordance rate in monozygotic twins (50%) [24].

However, some evidences have indicated controversial results in terms of environmental factors and ASD development. These studies suggest that environmental factors, such as vaccination, advanced parental age, maternal smoking, pregnancy and birth complications, thimerosal exposure, deficiency of vitamin D, and reproductive technologies have strongly correlated with ASD [16, 24, 25], but few studies showed no relation with ASD risk [24]. On the other hand, few studies also suggested that both genetic and environmental factors act in a synergistic manner, and epigenetic modifications could be mediators in the gene/environment interface [26, 27]. Various mechanisms have been proposed in such way, including DNA methylation, MECP2 mutation, folate-methionine pathway enzymes, histone acetylation, and chromosome remodeling for tracking of epigenetic changes [28].

Additionally, while few research groups reported that oxidative stress has been involved in ASD [29], also genetic abnormalities in glutathione (GSH) metabolism may be related to altered mitochondrial function [30]. Indeed, GSH, the key intramitochondrial reactive oxygen species (ROS) neutralizer, is decreased in ASD [31, 32]. Also, changes in glutamate metabolism have been implicated in ASD, with glutamate being postulated as a likely cause of mitochondrial dysfunction and selective Purkinje neuron degeneration stated in ASD [8].

## 3. From the Historical Perspective to Current Clinical Practice

Autism was first identified by Kanner [33] as an “inborn autistic disturbance of affective contact.” Then, many researchers tried to identify the definition and diagnostic criteria of autism [34]. The criteria for the diagnosis of childhood autism was established in 1993 [35]. Lobotomy was one of the initial ASD treatments [36, 37]. Psychotherapy [38] and holding therapy [39] were also practiced. The first medical drug that was approved to be used in autism was risperidone [40].

During the last decade, the frequency rate of the disease has been progressively rising [41]. Neurological dysfunctions such as autism substitute for a high impact in societies via the world. Though the indications resulting from those illnesses are popular, the mechanisms and reasons are composite and rely on multiple parameters [42]. ASD contains multiple dysfunctions with different stages of verbal skills, logical functioning, and several genetic etiologies [43].

Neurobiological systems being important for social functioning are debatably the most hopeful signaling pathways for ASD biomarker and therapeutic target detection. Today, two such nominees are the arginine vasopressin (AVP) and oxytocin (OXT) signaling pathways [44].

Apart from the earlier clinical studies, traditional medicine has been commonly used in ASD treatment [45]. Nevertheless, the negative effects of these medications or their adverse interaction between other medications are questionable, and the parents of the autistic children receiving treatment should be given information about the risks [46].

Japanese traditional herbal medicine, Kami-shoyo-san and Yokukansan, can be helpful in improving behavioral problems in autism [47, 48]. Moreover, Ayurvedic, Siddha, and homeopathic treatments are commonly used in India [49]. For instance, Panchagavya gritha was suggested as an effective Ayurvedic drug for autism [50]. In addition, acupuncture is also widely used in China [51, 52]. Positive effects of electroacupuncture on children with ASD were reported [53, 54].

It was suggested in several studies that ASD can be associated with nutritional and gastrointestinal problems, such as food selectivity, nutritional deficiencies, food allergies, intolerances, diarrhea, and constipation [55–59]. Thus, dietary approaches, including ketogenic diets, gluten-free and casein-free diets, high-fat diets, probiotic use, food additives, camel milk consumption, and other diets, have been frequently investigated [57]. Multivitamin/mineral supplementations are also frequently prescribed [60]. Some food components seem to cause both behavioral and gastrointestinal symptoms in children having ASD [61]. Autoimmunity may rise out of enhanced immune response to potential cross reactivity and dietary proteins to proteins in the brain or gut. Remarkably, children on specific protein-restricted diets display lower stages of activated underlying lamina propria lymphocytes (CD3+TNF-*α*+) known to be rich in immune and colonic intraepithelial lymphocytes compared to children on unrestricted diets [62].

When it comes to digestive capacity, if it is impaired in a subgroup of ASD children, another treatment option is the use of digestive enzyme supplementation, precisely with a full panel of protease enzymes, despite the limited number of researches carried out to assess the effectiveness of digestive enzyme supplementation on behavioral and gastrointestinal conditions [63].

On the one hand, although probiotic therapy has been advised in several reviews as a potential cure for children with ASD and gastrointestinal indications [64], it has been explained that as of 2009, only one-fifth of physicians were inspired to use probiotics in children with ASD [65]. One research showed that *L. acidophilus* management (5 × 109 CFU/day, twice a day for 2 months) expressively decreased a marker of offensive pathogenic candidiasis in children with ASD and gastrointestinal indications [66], although half of the children in the researches were on limited diets and the research design did not have a control group [66]. In 2014, Rossignol and Frye [67] systematically reviewed a large number of studies on Alzheimer's medications in ASD including donepezil, galantamine, rivastigmine, tacrine, and memantine. The results of some medications showed encouraging evidence for effectiveness against treating core and associated ASD symptoms, but clinical trials are also needed to confirm their efficacy for treating ASD individuals.

## 4. Bioactive Molecules: An Upcoming Key in Autism Spectrum Disease

Even though ASD is a lifelong neurodevelopmental disorder with no cure, there are some pharmacological treatments available to suppress symptoms, like irritability and suppression, and to treat other psychiatric problems that accompany ASD, such as depression, bipolar disorder, and anxiety [68]. Several researchers have reported that ASD is associated with nutritional disorders. Thus, the importance of diet and food consumption is huge in ASD treatment [69, 70]. Certain bioactive molecules have been suggested to support the treatment of autism. The effects of plant-based bioactive materials on autism are summarized in the following subsections.

### 4.1. The Role of Bioactive Molecules in Autism Spectrum Disease

**Figure 2:**
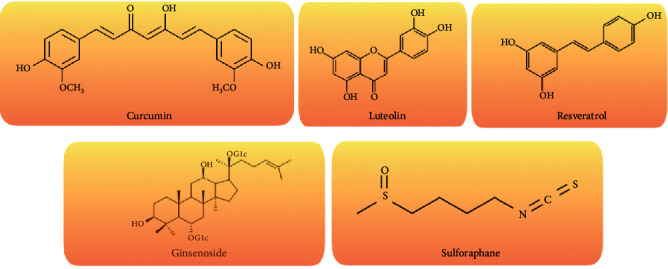
The chemical structure of some potential phytochemicals for ASD treatment.

Bioactive molecules that have neuroprotective effects can be used as natural agents in the treatment of neuropsychological disorders, such as ASD, depression, and bipolar disorder [42, 71, 72]. Curcumin, luteolin, and resveratrol are the frequently investigated plant-based active components (Figure 2).

Curcumin, which is a bioactive compound of turmeric (*Curcuma longa* L.), has the potential to be used in the treatment of neuropsychiatric disorders including autism [71]. Even though clinical trials are yet to come, there are a couple of promising studies on rodents. Bhandari and Kuhad reported that after curcumin treatment at a daily dose of 50, 100, and 200 mg/kg for 4 weeks in rats with propionic acid- (PPA-) induced autism, curcumin restored the core and associated symptoms of autistic phenotype by suppressing oxidative-nitrosative stress, mitochondrial dysfunction, and TNF-*α* and MMP-9 in PPA-induced autism in rats [73]. Curcumin could be developed as a potential pharmacotherapeutic adjuvant for ASD. In addition, Al-Askar et al. [74] reported the postnatal therapeutic role of curcumin in improving most of the impaired parameters in valproic acid- (VPA-) induced rodent models with persistent autistic features.

It was reported that luteolin can reduce maternal immune activation-induced neural abnormalities, such as ASD [75]. In addition, Asadi and Theoharides [56] reported that it can be utilized in ASD treatment, as luteolin inhibited mast-cell activation due to allergy augmentation and mitochondria stimulation. Moreover, dietary luteolin supplementation resulted in the reduction of serum interleukin-6 and tumor necrosis factor levels in children with ASD [76]. Dietary supplementation (1 capsule per 10 kg body weight) containing luteolin (100 mg/capsule), quercetin (70 mg/capsule), and rutin (30 mg/capsule) in olive kernel oil for 26 weeks enhanced adaptive functioning and behavioral disorders in children with ASD [77]. Moreover, Theoharides et al. [78] reported that a dietary supplement, NeuroProtek®, that contains luteolin, quercetin, and rutin, improved symptoms related to gut and brain inflammation in children (aged 4-12 y) with ASD.

Resveratrol has shown therapeutic and anti-inflammatory effects on neurological disorders [79]. For instance, prenatal resveratrol treatment of rats that were also given VPA during the prenatal stage to induce autism, with daily 3.6 mg/kg injections for 13 days, resulted in enhancement in behavioral changes with low interaction between resveratrol and VPA [80]. In another study, the restoration of the autism-related dysfunctions, such as neurological, behavioral, sensorial, biochemical, and molecular changes, were reported in rats with PPA-induced autism after daily oral resveratrol treatment of 5, 10, and 15 mg/kg for 4 weeks [81].

Korean red ginseng supplementation improved social interaction of mice with autism-like disorder [82]. Gonzales et al. [83] reported an improvement in autism-related behavioral problems with no effects on motor coordination ability due to a daily Korean red ginseng oral dose of 100 or 200 mg/kg in mice with VPA-induced autism (prenatal exposure).

Niederhofer [84] suggested that *Ginkgo biloba* can be added to the treatment of autism patients, as they observed improvement on the “aberrant behavior” and symptom checklist after *Ginkgo biloba* treatment of three patients. However, *Ginkgo biloba* extract was used in adjunction to standard autism medication, risperidone, for treatment of autistic children, and no significant effect was observed [85].

Isothiocyanates such as sulforaphane that is found in high amounts in broccoli sprouts has been found to ameliorate ADS symptoms [72]. Moreover, capsules of sulforaphane-rich broccoli sprout extracts (50 *μ*mol/capsule) were given to male patients with ASD (aged 13-30 y) in doses according to body weight (one capsule for <100 lb, two capsules for 101–199 lb, and three capsules for >150 lb), and improvement in ASD-related problems, such as oxidative stress, weakened GSH synthesis, lower mitochondrial function, decreased oxidative phosphorylation, lipid peroxidation, and neuroinflammation, were reported [86].

All these studies show that some natural plant-bioactive compounds can be promising tools in the control of ASD (Table 1).

Other formulations of bioactives can also be used in the treatment of autistic disorders. For instance, folinic acid (800 *μ*g oral powder in juice, twice a day), betaine (1000 mg oral powder in juice twice a day), and methylcobalamin (75 *μ*g/kg injectable liquid twice a week) improved the metabolic imbalance in autistic children [87]. Moreover, improvement in glutathione redox status of ADS children was observed after methylcobalamin (75 *μ*g/kg injected every third day) and folinic acid (400 *μ*g twice per day) treatment [88]. In another study, it was reported that ubiquinol improved ASD-related problems, such as behavior, eating, and sleep disorders in children (aged 3-6 y) [89].

Methylcobalamin and folinic acid have also been studied in combination with sapropterin, a synthetic form of tetrahydrobiopterin (BH4) [90]. These supplementations have shown therapeutic potential by affecting the folate, methylation, and glutathione pathways. There are also trials where supplementation with only BH4 has been investigated, where the treatment dose is 20 mg/kg/day of BH4. A trial with ASD children has demonstrated the significant improvements over placebo in social awareness, autistic gestures, hyperactivity, and inappropriate speech [91]. Other assays have shown that BH4 is involved in multiple metabolic pathways. It has been reported that this substance is involved in the improvement of the redox state, pterin, biogenic amines, nitric oxide, or inflammation [92–95].

### 4.2. Drifting from Natural to Synthetic Drugs

Plant-bioactive molecules demonstrated promising results in ASD treatment. However, the number of clinical trials is limited, and none of them have been approved for ASD or ASD-related disorder treatment. Thus, bioactive compounds and vitamin/mineral supplements are used as an adjunct to the synthetic drugs used to treat autism. The medications of autism generally target behavioral problems, developmental disorders, physiological destructions, and other cooccurring psychiatric and medical conditions, such as sleep and gastrointestinal problems, hypertension, and diabetes [96, 97].

Antipsychotic agents, such as risperidone and aripiprazole, are commonly used for treatment of ADS. DeVane et al. [98] reported that risperidone or aripiprazole treatments improved the Aberrant Behavior, Checklist-Irritability scores, behavior, and weight gain of patients with autistic disorder. The positive effects of risperidone on several problems associated with autism, such as Aberrant Behavior Checklist scores, and behavioral problems (e.g., aggression, explosivity, hyperactivity, irritability, repetition, self-injury, and social withdrawal) with a side effect of weight gain, were reported in several studies [99–103]. Aripiprazole also gave similar results in the treatment of ASD patients, such as improvements in behavioral disorders (hyperactivity, irritability, sleep problems, etc.) often with side effects, such as weight gain, and higher risk for sedation and tremor [104–107].

Although risperidone or aripiprazole are the only medications approved by the US Food and Drug Administration for ASD treatment [98, 108, 109], there have been several studies that some drugs developed to treat other mental disorders can be used in the treatment of ASD [67]. For instance, donepezil [110, 111], galantamine [112], and memantine [113], which were approved to treat Alzheimer's disease, were reported to improve autistic disorders, such as behavioral, communication, and social impairments. For instance, guanfacine developed for hypertension treatment was found effective in improving disorders in people with pervasive developmental disorders (PDD), such as ASD [114]. However, there have been several studies showing that metformin can be helpful in controlling weight gain in autistic patients [115–118].

Several other drugs are being investigated and new drugs are also being developed by scientists to treat autism. For instance, a single dose of suramin (20 mg/kg) improved behavioral problems due to autism and schizophrenia in mice models [119]. In addition, Naviaux et al. [120] reported that 20 mg/kg intravenous infusion (one dose) of suramin resulted in improvements in autism syndromes such as behavior and language in autistic children. Ong et al. [121] reviewed the use of phospholipase A2 inhibitors in the treatment of neurological disorders including autism.

Methylphenidate [122, 123], sertraline [124, 125], atomoxetine [126–128], and alpha agonists [129] were also reported to have potential to be used in autism treatment, even though they may have some side effects, such as mood change, irritability, and gastrointestinal disturbance.

Apart from central drugs such as the antidepressants or antipsychotics mentioned above, drugs such as propranolol, memantine, d-cycloserine, and oxytocin can be used [130]. One of the most important is propranolol, a beta-adrenergic antagonist that inhibits anger in higher doses improving various neuropsychiatric disorders [131, 132]. Trials have shown that propranolol improves altered emotional states that occur with anxiety, aggressiveness, self-harm, and hypersexuality [133].

Furthermore, a dysregulation in the hypothalamic-pituitary-adrenal axis and consequently in cortisol levels has been observed among people with autism spectrum disorder (ASD) [134]. Therefore, the regulation of steroid signaling has been suggested as a potential therapeutic route for the treatment of ASD and other disorders of the central nervous system [135].

Food selectivity and picky eating habits in patients with ASD are common, and the patient may also suffer from nutrient deficiencies even though the deficiency itself elevates the symptoms. Thus, food supplements are generally viewed as essential elements of ASD treatment. As vitamin and mineral deficiencies are common in ASD patients [55, 136–139], vitamin-mineral supplements are frequently investigated [140, 141]. Improvements in several ASD symptoms due to vitamin and mineral supplementations were reported [60, 142–145]. On the other hand, Stewart et al. [146] suggested that dietary supplementation, including vitamin and mineral supplements, should be done carefully, as they may not provide sufficient amounts of the deficient nutrient or may cause excessive intake. On the other hand, ASD patients are often treated with several drugs and food supplements at the same time, and the interactions between them are not well known and can be undesired [147]. In addition, further comprehensive investigations on the side effects of these medications are still required.

## 5. Plant-Food-Derived Bioactive Studies in Autism Spectrum Disease

### 5.1. In Vitro Studies

Mitochondrial function is critical to CNS as evident in its role in brain energy turn-over [148] and maintenance of ionic gradients critical to neurotransmission and plasticity [149], and its involvement in neural stem cell proliferation, differentiation, and maturation as well as formation of dendritic processes, developmental and synaptic plasticity, and cell survival and death [1, 150, 151] reinforces the importance of mitochondrial dysfunction in ASD etiology. Several phytochemicals have been revealed to protect or restore mitochondrial function and chief among them are the polyphenols with potent antioxidant properties.

Quercetin, a polyphenol broadly distributed in many plants and vegetables, exhibits strong antioxidant properties and could prevent oxidative stress. Research has shown that quercetin and epigallocatechin-3-gallate are bioaccumulated in the mitochondria in its active form, and this could be responsible for their mitochondria protective effect via ROS-scavenging mechanism in vivo [152, 153]. Studies have revealed the modulatory effect of polyphenols such as resveratrol, quercetin, and hydroxytyrosol on the mitochondrial biogenesis process via the stimulation of several coactivators and transcription factors such as the proliferator-activated receptor coactivator-1*α* (PGC-1*α*) [153]. Furthermore, several polyphenols have been shown to activate silent information regulation 2 homolog 1 (SIRT1) in vitro; thus, they have been increasingly searched as potential inducers of mitochondrial biogenesis through PGC-1*α* deacetylation-mediated activation [154]. Recently, resveratrol has been reported to stimulate the SIRT1/PGC-1*α*-dependent effect on mitochondrial biogenesis in cultured endothelial cells [155]. Furthermore, resveratrol has been revealed to positively influence mitochondrial performance in C2C12 cells via evoking AMP-dependent protein (AMPK) activation and increased mitochondria biogenesis [156]. Sulforaphane (isothiocyanate) preserved mitochondrial functions in ischemia- or toxin-induced damages in normal noncancerous cells [157] and also stimulated mitochondrial biogenesis [158]. Quercetin, rutin, and resveratrol have been shown to prevent the ATP drop and indomethacin-induced alteration in mitochondrial membrane potential in Caco-2 cells [159].

Glutamate dysregulation and toxicity have been observed in ASD, and there is ample evidence that phytochemicals could counteract the effect of altered glutamate metabolism. Polyphenols from green tea have been shown to attenuate glutamate excitotoxicity via antioxidative and antiapoptotic pathways in cultured cortical neurons [160]. In 2003, Lee et al. revealed the protective effect of polyphenols baicalin, baicalein, and wogonin isolated from *Scutellaria baicalensis* Georgi against glutamate/glucose-induced neurotoxicity in primary cultured rat central neurons via an increase in the cell viability, attenuation of increased intracellular calcium ions and nitric oxide production with baicalein being the most effective [161]. Furthermore, phlorofucofuroeckol isolated from brown algae species improves glutamate-induced neurotoxicity through modulation of oxidative stress-mediated mitochondrial dysfunction in PC12 cells [162].

All these in vitro studies with plant-food-derived bioactives in autism spectrum disease are summarized in Table 2.

### 5.2. In Vivo Studies

Given the rapidly growing disabilities triggered by ASD, characterized by social deficits, communication impairment, and cognitive flexibility deficits, McKinnell et al. evaluated in 2021 the changes in rodent behavior (social and anxiety) and cognitive flexibility in the VPA model of autism and control. The results indicated that VPA rats showed loss in performing the set-shifting task. In other words, females with ASD displayed unique behavioral profiling compared to males with ASD [163]. In 2021, Rebolledo-Solleiro et al. examined the effect of bisphenol A (BPA) on behavior, neurodevelopment, and neurodegeneration through a systematic review. As their main findings, the authors underlined that BPA modulates the normal functioning of the reproductive system, metabolism, and brain functions, while triggering the development of few neurodevelopmental disorders including ASD [164].

**Figure 3:**
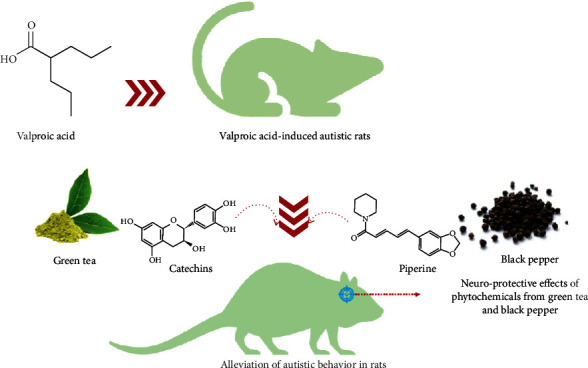
Positive effect of plant extracts (green tea and black pepper) in the management of autistic behavior in rat model.

Similarly, an increasing amount of naturally occurring bioactive compounds have been used for ASD management. Among them, *Camellia sinensis* (green tea) is an important dietary source of polyphenols, specifically flavonoids, with catechins, such as epigallocatechin-3-gallate, epigallocatechin, epicatechin-3-gallate, and epicatechin being the dominant ones. However, the presence of gallic, chlorogenic, and caffeic acids and flavonol derivatives, like kaempferol, myricetin, and quercetin have been stated [165, 166]. Significant improvement in behavioral assessments and neuroprotection and lowered oxidative stress were observed in autistic mice treated with flavonoid extract from green tea [166]. Furthermore, histological findings revealed the presence of a distinct Purkinje layer and cells after treatment with green tea, thus suggesting its neuroprotective effect [166]. The positive effect of plant extracts (green tea and black pepper) in the management of autistic behavior in rat models is illustrated in Figure 3.

Also, piperine, the major alkaloid found in *Piper longum* L. and *Piper nigrum* L. (black pepper), has been reported to possess antioxidant, neuroprotective, anxiolytic, and cognition-enhancing effects [167–169]. A previous study has revealed ameliorative effects of piperine on behavioral alterations and oxidative stress markers in ASD murine model as evident by improved/restored motor deficits and decreased reorientation time, due to its capability to mitigate sodium valproate-induced cerebellar damage [170]. Piperine treatment also brought on cerebellum integrity restoration via a decrease in Purkinje cell number, which is connected with the cerebral cortex and the limbic system [170]. For these properties and anti-inflammatory, antioxidant, and neuroprotective effects, resveratrol could also be relevant in ASD treatment [171, 172].

A study in an experimental murine model revealed that resveratrol improves social skills in valproic acid-induced autistic rats. It regulates and activates sirtuins, members of the class-III histone deacetylases, and also exerts neuro-immunomodulatory effects by regulating transcription factor signaling, decreasing proinflammatory molecules (IL-6 and TNF-*α*) on dopaminergic neurons, inhibiting NF-*κ*B activation, and suppressing T cells [80, 173]. Another study reported that resveratrol suppresses neuroinflammation, mitochondrial dysfunction, oxidative/nitrosative stress, and TNF-*α* expression in propanoic acid-induced autistic rats [81], thus making it a potential therapeutic agent to ameliorate ASD's neurobehavioral, and biochemical changes [81].

The impact of turmeric on neurodegenerative diseases and neuropsychiatric disorders has also been documented. Curcumin (diferuloyl methane), the major curcuminoid present in turmeric, a relatively nontoxic and permeable compound to the blood-brain barrier [174, 175], has been reported to have positive effects on the treatment of autistic rats as it targets several cell signaling pathways. For instance, it increases intracellular GSH levels, reduces inflammatory components, and mitigates mitochondrial dysfunction and oxidative/nitrosative stress as well as protein aggregation [73, 176]. This study has also shown that curcumin can alleviate autistic phenotype-associated symptoms via suppressing oxidative-nitrosative stress and mitochondrial dysfunction in propanoic acid-induced autistic rats [73]. In addition, curcumin is able to ameliorate delayed brain maturation and brain toxicity in an autistic animal model via restoration of altered neurological, behavioral, biochemical, and molecular changes related to ASD phenotype [73, 74].

Bacosides are medicinal substances widely used by Indian tribes and are the main bioactive compounds extracted from *Bacopa monnieri* (L.) Wettst [177]. This plant is traditionally known for its intellect- and cognition-improving properties as well as being a nerve tonic [178, 179]. The pharmacological properties of *B. monnieri* have been attributed to its constituent alkaloids, saponins, and sterols [180]. *B. monnieri* has significantly improved behavioral alterations, decreased oxidative stress markers, reduced pain threshold, and normalized locomotor deficiencies as well as anxiety in a murine model of autism. The improvement in locomotive activity was attributed to the antianxiety properties of *B. monnieri* and its ability to decrease accumulated glutamate and restore cerebellum architecture [181].

Long-time, daily oral administration of ginsenoside-rich extract has been found to improve social interaction, repetitive behaviors, locomotor activity, and other ASD-related behaviors in mice models [83]. Thus, ginsenoside may be viewed as a possible drug candidate for ASD-associated phenotypes and symptom management as well as neurobehavioral deficits. Ginsenosides are Korean red ginseng (*Ginkgo biloba* L.) phytoconstituents known for their therapeutic properties, such as improvement in cerebral blood flow, stimulation of neuronal plasticity, learning, memory, and cognition improvement as well as CNS-associated disease treatment [182–184]. In addition, this plant has been reported to possess antistress, neuroprotective, anti-inflammatory, and antioxidant properties [85, 185].

Furthermore, the effect of the ultramicronized lipid molecule, N-palmitoylethanolamide (PEA) with luteolin (co-ultra-PEA-LUT), in an ASD murine model revealed a reduction in proinflammatory molecules, such as nitrotyrosine and nuclear factor kappa B (NF-*κ*B), an improvement in neuroplasticity and neurogenesis, and the modulation of the apoptotic mechanism in several brain regions (cerebellum and hippocampus) following treatment with co-ultra-PEA-LUT [186]. Another study evaluated PEA on the autistic behavior of BTBR T+tf/J mice and shed light on the contributing mechanisms [187]. PEA improved the behavioral phenotype of BTBR mice dependent on PPAR-*α* activation. PEA restored the hippocampal BDNF signaling pathway and mitochondrial dysfunction and reduced the general inflammatory status of the mice, reducing the expression of proinflammatory cytokines at the hippocampal, serum, and colonic levels. It also improved intestinal permeability by increasing the expression of the tight junctions of the colon and the composition of the intestinal microbiota.

All these in vivo studies with plant-food-derived bioactives in ASD are summarized in Table 3.

PEA: N-palmitoylethanolamide; co-ultra-PEA-LUT: ultramicronized N-palmitoylethanolamide with luteolin.

### 5.3. Clinical Studies

Given the huge impact of ASD on the health, wellbeing, and quality of life of patients and facing the increasing evidences of the role of diet in both prevention and management of several diseases, Hartman and Patel reviewed a large number of reports on food approaches to the ASD management and their relation with gastrointestinal, behavioral, neurological, and immune functions through food supplements (fatty acid and pro- and prebiotics, vitamins, minerals, phytochemicals, and hormones) [188]. As their main statements, gastrointestinal issues were correlated with a number of behavioral and neurological deficits, with food approaches being able to improve the lives of patients with ASD [188]. In another study, Gogou and Kolios reported on the impact of food administration during pregnancy on the risk of ASD offspring. In this study, authors included both clinical and experimental studies, and food supplement (i.e., folic acid, iron, vitamins, choline, vitamin D, and docosahexaenoic acid) studies were also included [189]. Food supplements including choline, folic acid, and multivitamins had a significant impact on the expression of ASD-related genes, while iron had no effect. Thus, a suitable amount of multivitamins, vitamin D, and docosahexaenoic acid can help in reducing the risk of ASD in offspring [189]. Infante et al. reported a case study of a 23-year-old young adult male with ASD. Omega-3 and vitamin D combination therapy showed beneficial effects to the patient [190]. Also, Sivamaruthi et al. assessed the role of the microbiome, food supplements, and probiotics in the development of ASD and underlined that the maternal diet and lifestyle are greatly associated with the development of ASD [191].

In 2017, Wink et al. described the long-term impact of metformin on antipsychotic-associated weight gain in youth with and without ASD using brief ASD mealtime behavior inventory (BAMBI) and the behavior pediatric feeding assessment scale [118]. The authors stated that metformin treatment stabilized BMI *z*-score, and BAMBI demonstrated good consistency, test-retest reliability, and criterion-related reliability. In 2001, Ahearn et al. reported on the feeding behavior in children (ages 3–14 years; exposed to 12 food items in 6 sessions) with ASD and pervasive developmental disorder-not otherwise specified (PDD-NOS) [192]. Different parameters, including food acceptance, expulsion, and disruptive behavior were recorded on a trial-by-trial basis, with data obtained clearly indicating that some children were sensitive to food texture or category, while others were indifferent.

In addition, and when looking at clinical evidence reporting the use of plant-derived bioactive molecules on ASD, the anti-inflammatory, antioxidant, antiallergy, and neuroprotective effects of different biomolecules have been well documented. Mostafavi and Gaitanis analyzed a data set including preclinical and clinical data regarding the use of cannabis and cannabidiol in the treatment of ASD [193]. The results of the analysis suggested that both compounds revealed promising therapeutic benefits in some persons with ASD.

In the case of specific compounds, luteolin and quercetin extracted from *Chamomile* sp. and *Sophora* sp. leaves were used in children with ASD, and marked improvements in ASD symptoms were stated. Most of the patients (75%) reported a significant improvement in gastrointestinal features, such as in stool shape, smell, form, and color. In about 50% of children, habits were improved within a period of 2 to 3 weeks and “allergic-like” skin symptoms were also markedly reduced. Eye contact and attention also improved in about 50% of patients. In addition, 30%–50% of patients showed learned tasks and social interactions and about 10% of children started speaking words or short sentences. However, no improvements were recorded for hyperactivity or aggressiveness [194]. In 2013, Taliou et al. reported an improvement in adaptive functioning and in overall behavior of about 26.6%–34.8% of the autistic children when treated with luteolin, quercetin, and rutin extracted from *Chamomile* sp. and *Sophora* sp. leaves [77]. In another clinical study, co-ultra-PEA-LUT led to an improvement in ASD-related symptoms and reduced motor stereotypes [186]. Another study with PEA showed beneficial effects of this treatment in two child patients with ASD [195]. These subjects showed significantly improved cognitive aspects, expressive language, and sociability, as well as the general severity of autism. Furthermore, slight improvements were observed in irritability, hyperactivity, eye contact, and speech following treatment with a flavonoid component extracted from *G. biloba* leaves [84].

All these clinical studies with plant-food-derived bioactives in ASD are summarized in Table 4.

## 6. Conclusions and Upcoming Perspectives

Plant-bioactive molecules demonstrated promising results in ASD treatment. However, there is a scarce number of clinical trials available so far, and none of them have been approved for the treatment of ASD or ASD-related disorder. Thus, further preclinical and clinical studies are needed for a more in-depth understanding of plant-derived bioactives as drug discovery candidates on ASD treatment.

## Figures and Tables

**Table 1:** Protective action of bioactive compounds from plants in the management of ASD.

Phytocompounds	Dose	Treatment of ASD behavior	References
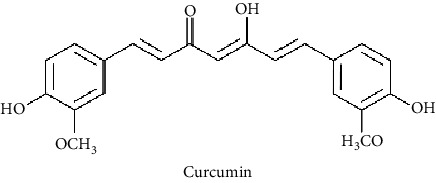	Curcumin: 50, 100, and 200 mg/kg for 4 weeks	Neurological, behavioral, biochemical and molecular changes lead to management of ASD	[71, 73, 74]
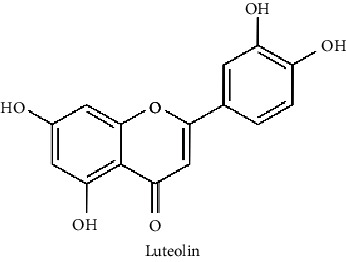	Luteolin: 100 mg/capsule Quercetin: 70 mg/capsule Rutin: 30 mg/capsule For 26 weeks	Improved adaptive functioning and behavioral disorders for managing ASD	[56, 75–78]
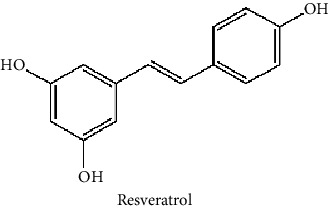	Resveratrol: 3.6 mg/kg for 13 days	Improved neurological behavioral, sensorial, biochemical, and molecular changes leading to the management of ASD	[79–81]
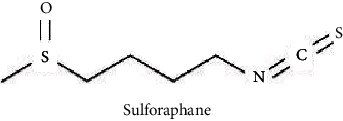	Sulforaphane: 50 *μ*mol/capsule	Successful management of ASD	[72, 86]

**Table 2:** Summary of in vitro studies.

Phytocompounds	In vitro model	Results	References
Quercetin	Jurkat cells	Antioxidant properties Mitochondria protective effect	[152, 153]
Resveratrol	Endothelial cells	Stimulate SIRT1/PGC-1*α* and increase of mitochondria biogenesis	[155]
Resveratrol	C2C12 cells	AMPK activation and increase of mitochondria biogenesis	[156]
Sulforaphane	Ischemia induced in normal noncancerous cells	Preserve mitochondrial functions and increase of its biogenesis	[157, 158]
Quercetin, rutin, and resveratrol	Indomethacin-induced Caco-2 cells	Prevent the ATP	[159]
Green tea polyphenols	Cortical neurons	Antioxidative and antiapoptotic properties	[160]
Baicalin, baicalein, and wogonin isolated from *S. baicalensis*	Primary culture rat central neurons	Antioxidative properties, increased cell viability, reduced intracellular calcium ions and nitric oxide production	[161]
Phlorofucofuroeckol	Glutamate-induced toxicity in PC12 cells	Improvement of mitochondrial dysfunction	[162]

**Table 3:** Summary of in vivo studies.

Phytocompounds	In vivo model	Results	References
Bisphenol A	Different ASD animal models	Modulates the function of the reproductive system, metabolism, and brain functions	[164]
Green tea	ASD mice model	Neuroprotective and antioxidant properties, and improvement of behavior	[166]
Piperine	ASD murine model	Antioxidant, neuroprotective, anxiolytic, and cognition-enhancing effects	[167–169]
Resveratrol	VPA-induced ASD rats	Activates sirtuins and decreases IL-6, TNF-*α*, NF-*κ*B, and T cells	[80, 173]
Resveratrol	Propanoic acid-induced ASD rats	Reduces neuroinflammation, mitochondrial dysfunction, and oxidative/nitrosative stress	[81]
Curcumin	ASD rats	Increases GSH levels and reduces inflammation, mitochondrial dysfunction, and oxidative/nitrosative stress	[73, 176]
*B. monnieri*	ASD murine model	Improvement of behavior and antioxidant, anxiolytic, and analgesic properties	[181]
Ginsenoside-rich extract	ASD mice model	Improvement of behavior and locomotor activity	[83]
Co-ultra-PEA-LUT	ASD murine model	Reduces proimflammatory markers (nitrotyrosine and NF-*κ*B), improves neuroplasticity and neurogenesis, and modulates apoptosis	[186]
PEA	BTBR T+tf/J mice	Reduces mitochondrial dysfunction and inflammatory effects Improvement of behavior, PPAR-*α* activation Microbiota-gut-brain axis regulation	[187]

**Table 4:** Summary of clinical studies.

Phytocompounds	Subjects	Results	Reference
Food supplements including choline, folic acid, and multivitamins	Pregnant patient	Reduced expression of ASD-related genes	[189]
Omega-3 and vitamin D	Case study of ASD patient	Reduced ASD symptom	[190]
Metformin	ASD adult patients	Stabilized BMI *z*-score	[118]
Cannabis and cannabidiol	ASD adult patients	Reduced ASD symptom	[193]
Luteolin and quercetin	Children with ASD	Improvement in gastrointestinal features, eye contact, and attention social interactions	[194]
Luteolin, quercetin, and rutin	Children with ASD	Improvement in adaptive functioning and in overall behavior	[77]
Co-ultra-PEA-LUT	ASD adult patients	Reduced motor stereotypes, anxiety, and worsening in social skills	[186]
PEA	Children with ASD	Improvement of cognitive aspects, expressive language, and sociability Reduced general severity of autism	[195]
Flavonoid component extracted from *G. biloba*	ASD adult patients	Slight improvements in irritability, hyperactivity, eye contact, and speech	[84]
